# Using Knowledge Fusion to Analyze Avian Influenza H5N1 in East and Southeast Asia

**DOI:** 10.1371/journal.pone.0029617

**Published:** 2012-05-17

**Authors:** Erjia Ge, Robert Haining, Chi Pang Li, Zuguo Yu, Miu Yee Waye, Ka Hou Chu, Yee Leung

**Affiliations:** 1 Department of Geography and Resource Management, The Chinese University of Hong Kong, Hong Kong, China; 2 School of Public Health and Primary Care, The Chinese University of Hong Kong, Hong Kong, China; 3 Department of Geography, University of Cambridge, Cambridge, United Kingdom; 4 School of Life Sciences, The Chinese University of Hong Kong, Hong Kong, China; 5 School of Mathematics and Computational Science, Xiangtan University, Hunan Province, China; 6 School of Biomedical Sciences, The Chinese University of Hong Kong, Hong Kong, China; 7 Institute of Environment, Energy and Sustainability, The Chinese University of Hong Kong, Hong Kong, China; Centro Nacional de Microbiología - Instituto de Salud Carlos III, Spain

## Abstract

Highly pathogenic avian influenza (HPAI) H5N1, a disease associated with high rates of mortality in infected human populations, poses a serious threat to public health in many parts of the world. This article reports findings from a study aimed at improving our understanding of the spatial pattern of the highly pathogenic avian influenza, H5N1, risk in East-Southeast Asia where the disease is both persistent and devastating. Though many disciplines have made important contributions to our understanding of H5N1, it remains a challenge to integrate knowledge from different disciplines. This study applies genetic analysis that identifies the evolution of the H5N1 virus in space and time, epidemiological analysis that determines socio-ecological factors associated with H5N1 occurrence, and statistical analysis that identifies outbreak clusters, and then applies a methodology to formally integrate the findings of the three sets of methodologies. The present study is novel in two respects. First it makes the initiative attempt to use genetic sequences and space-time data to create a space-time phylogenetic tree to estimate and map the virus' ability to spread. Second, by integrating the results we are able to generate insights into the space-time occurrence and spread of H5N1 that we believe have a higher level of corroboration than is possible when analysis is based on only one methodology. Our research identifies links between the occurrence of H5N1 by area and a set of socio-ecological factors including altitude, population density, poultry density, and the shortest path distances to inland water, coastlines, migrating routes, railways, and roads. This study seeks to lay a solid foundation for the interdisciplinary study of this and other influenza outbreaks. It will provide substantive information for containing H5N1 outbreaks.

## Introduction

Highly pathogenic avian influenza (HPAI) H5N1 first isolated in Guangdong province, southern China in 1996 [1]. The HPAI H5N1 virus was redetected in East-Southeast Asia in late 2003 associated with large outbreaks among poultry in Thailand, Vietnam, Indonesia, and China [2]. The HPAI H5N1 influenza virus has established multiple regional sub-lineages and shown long-term persistence in these countries [3]. Also, the virus has continued its geographical migration through Southeast Asia to Eurasia and Africa. Disease control measures resulted in large numbers of domestic and wild birds being killed or culled to prevent a possible avian-influenza pandemic that might cost still more human lives in addition to huge economic loss [4]. HPAI H5N1 is considered a serious public health risk despite relatively low numbers of human infections. The public health risk arises through the high human mortality rates (to September 2010, a total of 505 patients from 15 countries were known to have been infected by H5N1, of which about 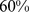 had died [5]) and the prospect that the H5N1 virus might reassort with another influenza A strain to produce a highly pathogenic and transmissible virus in humans [6]. Moreover, the wide distribution of H5N1 in South-East Asia provides ample opportunity for reassortment [7]. Identifying areas that are at risk of H5N1 is important for the development of strategies to control this disease.

Traditional genetic analysis, which infers the phylogenetic relationships associated with the H5N1 virus from its genomic sequences, has enabled progress to be made in understanding the evolution of avian influenza viruses [2], [8]. By this means, possible sources and pathways that are associated with the spread of the H5N1 virus can be inferred [8], [9]. Phylogeographic analysis [10] offers a method of tracking the migration of the H5N1 virus. It analyzes the topology of the phylogenetic tree and uses evolutionary models to statistically infer the resident localities of the H5N1 virus [11]. Medical geography examines the spatial pattern of H5N1 looking for localized hot spots where outbreaks are significantly clustered [12]. In spatial epidemiology, risk factor analysis focuses on the identification of factors associated with H5N1 occurrence and statistical modeling is used to predict the incidence of the disease [13]. All these different forms of analyses have a common objective which is to understand the distribution and the spread of avian influenza. The study of avian influenza H5N1 is multi-disciplinary across Virology, Molecular Biology, Evolutionary Biology, Medical Geography, and Spatial Epidemiology.

However, any study that relies on only one kind of disciplinary knowledge may miss important connections. For example, not taking into account the effects caused by geographical scale (the so-called “modifiable areal unit problem” [14]) could lead to interpretation errors when reviewing results from statistical analysis and modeling in area-based epidemiology. The predictive mapping of H5N1 risk in China, for instance, in Fang et al. [15], conflicts with empirical observations [5], [16] and previous studies [17], [18]. Also, phylogenetic analysis [19] has its own limitations when genetic data are incomplete, and evolutionary models inappropriate [20]. This form of analysis, which provides a micro-scale insight into the process of viral evolution, is insufficient for understanding macro-scale spread of avian influenza. In addition, uncertainty, perhaps arising from incomplete data, limited domain knowledge, or the application of an insufficiently sophisticated methodology could limit the value of these analyses. Wallace et al. states that limited sampling may lead to results that are not statistically significant [11].

Although current research tends to integrate multi-disciplinary studies of avian influenza, it stops at the early stage of analytically integrating data, for example, on phylogenetic relationships between isolated occurrence of the virus, migratory bird movements, and trade in poultry and wild birds [21], [22] or implementing only basic statistical analysis between genetic distance and geographic distance [23]. Even though knowledge has been acquired in many different disciplines, adequate methods to quantify and integrate this knowledge for H5N1 remain poorly developed.

This article proposes a novel approach to integrating the findings of phylogenetic analysis, which unravels H5N1 evolution in space and time, with modified local *K* function analysis, which identifies outbreak clusters in space, and also with spatial epidemiology, which determines socio-ecological factors associated with the occurrence of H5N1. In this study, Dempster-Shafer theory of evidence [24], [25], a mathematical method for making inferences based on multiple forms of evidence and which recognizes the uncertainties associated with the different sources of evidence, is used to formally integrate the three sets of findings. The present study is novel and significant in seeking to lay a solid foundation for the inter-disciplinary study of this and other relevant influenza epidemics. First, it uses genetic sequences and space-time data to create a phylogenetic tree to estimate the virus' capability of spreading. This is the first attempt to provide a mapping of H5N1 viruses derived from the phylogenetic tree. Second, by integrating the results obtained from the three analyses, we offer insights into the occurrence and space-time spread of H5N1 that have a higher level of correlation with empirical evidence than is found when analysis is based on only one methodology. In addition, we apply the methodology across multiple scales; that is to the whole of East-Southeast Asia as well as the individual countries of Thailand, Vietnam, Indonesia, and China, respectively. Our analysis results in a significant advance in findings over those reported in, for example, Gilbert et al. [13], and we believe our findings are more precise and informative in representing the occurrence and the space-time dynamics associated with the spread of H5N1.

## Results and Discussion

### Thailand and Vietnam

**Figure 1:**
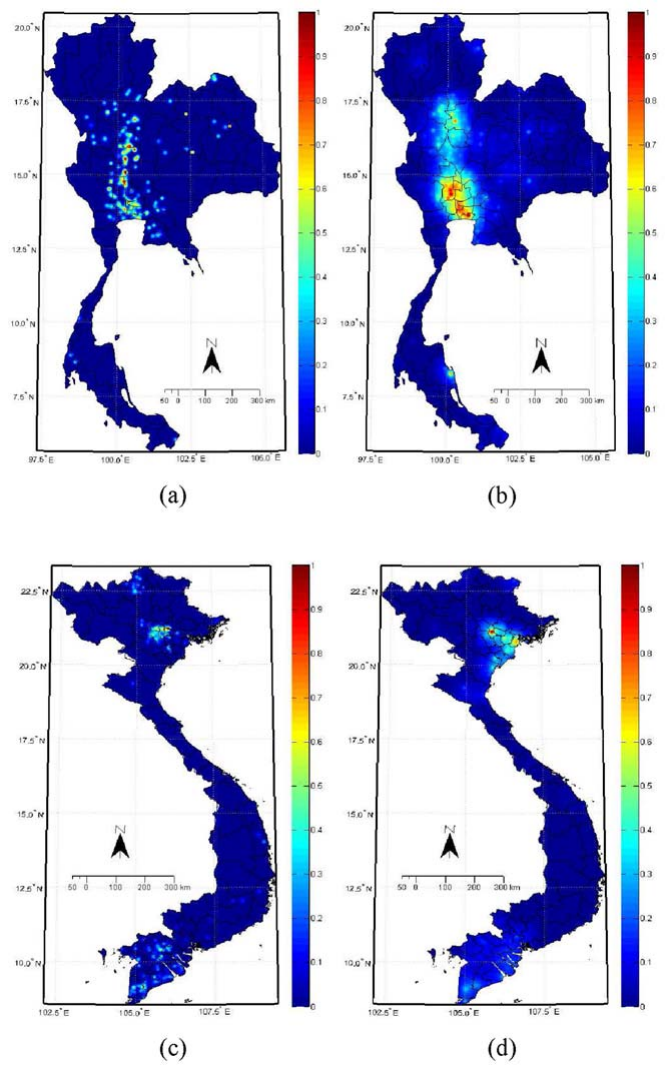
Probability maps predicting the occurrence of avian influenza (H5N1) in Thailand and Vietnam. (a) and (c) show the probabilities derived from the phylogenetic trees analyses (see Figure S3(b)); (b) and (d) show the results of the modified local *K* function analysis, depicting the spatial distribution of outbreak clusters. The experimental data covers the H5N1 outbreaks from late 2003 to 2009.

Figure 1 and Figure S1 show the result for Thailand and Vietnam. Figure S1(c) and (f) demonstrate our estimate of the spatially varying ‘degree of belief’ in the level of risk of H5N1 obtained by integrating the three forms of analysis (see Figures 1(a) and (c), and Figures 1(b) and (d), and Figures S1(b) and (e)) using the Dempster-Shafer theory of knowledge fusion. Figures S1(b) and (e) are the results from the epidemiological analysis of Gilbert. et al. [13]. In both cases the closer any area's value is to 1 (the redder it is) the greater the likelihood of an H5N1 outbreak in that area. Figure S1 also reports Pearson correlation coefficients (R) and associated *p*-values which show that our experimental patterns ((c) and (f)) have a closer correspondence to the observed pattern of cases ((a) and (d)) than the results of Gilbert et al. [13] ((b) and (e)). This finding holds over a range of spatial scales from 60
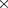
60 cell aggregates to 30
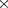
30 cell aggregates.

Figure S1(c) shows that the greatest risk of H5N1 is in the upper central region and the lower part of northern Thailand. It indicates less risk in central Thailand than predicted by Gilbert et al. [13] (Table 1(b)). This pattern corresponds with fewer observed outbreaks in this area of Thailand.

**Table 1:** Summary results of the logistic regression model for the avian influenza H5N1 epidemics in East-Southeast Asia, Indonesia, and China, 1996–2009.

Regent or Country	Con	Alt	PopDen	PolDen	D2Wat	D2Coast	D2Flyway	D2Rail	D2Road
East–Southeast Asia	.5371	−.001	.0007	−4.01 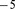	−.0037	−.0008	−.0007	−.0004	−.249
		p 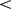 .001	p 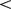 .001	p = .142	p 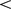 .001	p 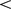 .001	p 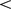 .001	p 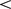 .001	p 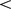 .001
Indonesia	−1.841	3.654 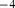	4.298 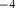	4.142 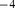	5.597 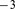	−4.383 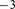	3.904 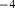	−1.853 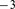	−.014
		p = .074	p 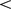 .001	p 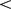 .001	p = .030	p = .012	p = .011	p 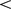 .001	p = .082
China (1996–2009)	−1.637	3.065 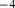	8.289 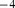	−4.74 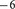	−.012	−2.458 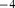	7.951 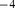	−9.076 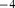	−.025
		p = .036	p = .004	p = .580	p 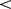 .001	p = .094	p = .003	p = .166	p = .012
China (1996–2004)	−1.367	6.515 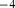	1.407 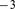	−4.947 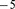	−.034	−3.718 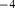	1.353 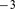	−4.431 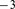	−.041
		p = .074	p = .078	p = .459	p = .0002	p = .288	p = .028	p = .049	p = .175
China (2005–2009)	−1.422	2.547 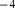	6.517 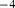	−3.429 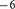	−.014	−4.7 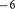	4.184 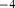	−7.944 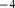	−.027
		p = .145	p = .083	p = .621	p = .001	p = .721	p = .169	p = .289	p = .020

These values are the average of 1000 bootstrap replicates of the logistic regression model. The meaning of the abbreviation shows as follow: Alt = average altitude; PopDen = population density; PolDen = poultry density, D2Water = minimal distance to inland water bodies; D2coast = minimal distance to coastline; D2Flyway = minimal distance to migratory bird pathways; D2Rail = minimal distance to railways; D2Road = minimal distance to roads. Con is the constant of the logistic regression models.

For Vietnam, the greatest risk occurs in the north and to a lesser extent the south of the country (Figure S1(f)). Unlike the work of Gilbert et al. [13], our results not only model the spatial distribution of H5N1 outbreaks, but also the space-time dynamics of viral evolution. Our analysis combines real-world outbreak data with evidence on viral evolution. For instance, the H5N1 virus was first detected and became established around Hanoi, north Vietnam, in 2001 and consequently spread to the south around Ho Chi Minh city [26]. Phylogenetic analysis shows that the virus, isolated from the north, has multiple sublineages and shares a close phylogenetic relationship with the virus from Thailand, Malaysia, Laos, and provinces in southern China [3], [8]. Furthermore, the northern H5N1 virus is associated with novel genetic subtypes, and these have facilitated the spread of the disease both within and outside the country [26]. Finally, it was reported that the number of H5N1 outbreaks decreased in the south in late 2005, but the disease still persists in causing outbreaks in northern Vietnam [16], [26]. Figure S1 again demonstrates that the risk estimates obtained from our integrated analysis correspond more closely than the results from Gilbert et al. to the empirical outbreak pattern at a range of different scales.

### Indonesia, China, and East-Southeast Asia

**Figure 2:**
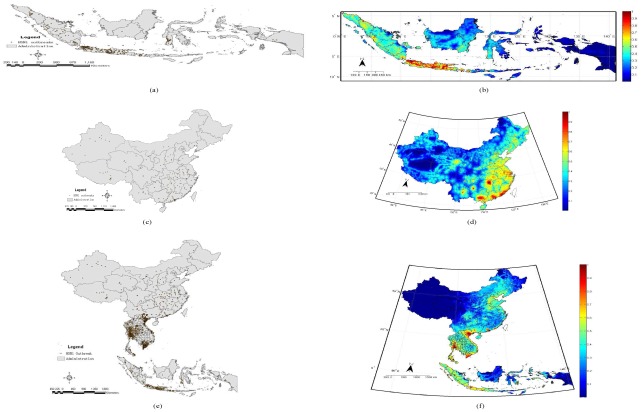
The spatial pattern of H5N1 in Indonesia, China, and East-Southeast Asia. (a), (c), (e) show the distribution of observed H5N1 outbreaks; (b), (d), and (f) show the probability maps integrating the findings of the phylogenetic analysis ( Figures S2(a), (d), and (g)), the modified local *K* function analysis (Figures S2(b), (e), and (h)), and the logistic regression analysis (Figures S2(c), (f), and (i)). The closer the probability is to 1, the greater is the probability of an H5N1 outbreaks.

Figure 2 shows the observed distribution of H5N1 outbreaks and our estimates of the risk of the disease in Indonesia, China, and the whole of East-Southeast Asia. Table 1 summarizes the logistic regression analysis in the three areas.

For Indonesia, the highest risk of H5N1 is in central Java (Figure 2(b)). It shows that the risk extends through the island to its surrounding archipelagos. The findings from the logistic regression analysis provide an interpretation for this pattern. Table 1 illustrates that population density, poultry density, and the shortest path distance to railways are significantly (*p* 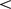.001) associated with occurrences of the disease. The results indicate that the very high density of population (more than 940 people per km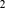) and the high concentration of poultry production (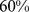 of Indonesia's production takes places in Java) are underlying factors in the establishment of multiple H5N1 subgroups [8]. These factors greatly increase the risk of H5N1 occurrence in Java. In addition, the significant negative relationship between H5N1 and the shortest path distance to railways implies that avian influenza is spread to other surrounding archipelagos through the production and trading of poultry [8].

For China, our result shows that the highest risk of H5N1 is in Guangdong, Yunnan, Fujian, and areas close to Dongting Lake in Hunan province, south China. High risk also appears to extend along the coastline of southern China (see Figure 2(d)). The risk increases from the northwest to southeast. Qinghai Lake, where over 6000 migratory birds were infected and killed by HPAI H5N1 in early 2005 [27], [28], was also highlighted as a potential source for the disease. The logistic regression analysis identified population density and the shortest path distance to inland water bodies as significantly (*p* 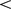.005) associated with the occurrence of H5N1 (Table 1). In eastern China, farming, free-grazing poultry, and economic trade and movement, have played significant roles in the maintenance and the transmission of avian influenza. The significant positive relationship (*p* 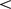.005) between the shortest path distance to migratory bird pathways and outbreaks, however, appears to suggest that bird migration may not be a key factor triggering large outbreaks and viral transmission in China, particularly in eastern China between 1996 and 2009, even though bird migration has been widely thought as a cause of wide spread of the disease globally [27]–[29]. The other socio-environmental variables (as shown in Table 1), including altitude, population density, poultry density, and the shortest distances to inland water bodies, coastlines, migratory bird pathways, railways, and roads, fail to show statistical significance within the two periods between 1996 and 2004, and 2005 and 2009. This might be due to data limitations associated with using such a short time span.

Figure 2(f) depicts the risk of occurrence of avian influenza H5N1 in East-Southeast Asia. The large-scale mapping shows that the highest risk is in central Thailand and the northern and southern parts of Vietnam. The central part of Indonesia also has high risk. Compared to these countries, China appears to have lower levels of risk, especially in the northwest including Tibet and Xinjiang autonomous regions, and the northern part of Qinghai province. This pattern can be regarded as a reflection of the relationship between the disease and heterogeneity of the local socio-ecological environment. The logistic regression analysis indicates that outbreaks significantly (*p* 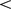.001) associated with altitude, population density, and the shortest path distance to inland water bodies, coastlines, railways, and roads (Table 1). In East-Southeast Asia, most cities and countries are usually located in areas where the environment is suitable for human habitation and agricultural production. Rice cropping and poultry rearing are popular in Thailand, Vietnam, Indonesia, and south China. These countries benefit from abundant hydrological resources (e.g., river deltas), but suffer from ecological-environmental problems caused by a rapidly increasing population which in turn facilitates the establishment of multiple H5N1 sublineages [27]. In addition, convenient transport networks like railways and roads become significant in spreading and triggering the re-occurrence of H5N1 in East-Southeast Asia.

## Materials and Methods

Three analyses are implemented on raster data frames. The probability of the occurrence of HPAI H5N1 is estimated for each lattice point (or small pixel). Specifically, this study consists of four parts. First, phylogenetic tree are built for the evolution of the H5N1 virus. The branches and the topology of the tree describe the processes of viral evolution. The ability of a virus to survive in nature (its “capability”) is a characteristic of a virus which goes through a long evolutionary process with wide spatial dispersion and persistence over time. Strong capability may lead to a high probability of the disease spreading widely. In this study, the years and localities from which the genetic sequences were sampled are collated for each H5N1 virus. Integrating in space and time, the quantification of the phylogenetic tree is a feasible way to measure and map the capability of the H5N1 virus. Second, the local *K* function, a spatial point pattern statistic [30], is modified for the purpose of identifying the local pattern of outbreaks. The estimates obtained from the analysis are an indicator of outbreak clusters. Third spatial epidemiological analysis involves building a logistic regression model for analyzing the statistical association between the presence/absence of reported H5N1 outbreaks and eight socio-environmental variables. This model can be used to predict the probability of the occurrence of an H5N1 outbreak. The findings obtained from the three analyses provide evidence with which to explore the spatial distribution of H5N1. To present a powerful, robust, and unified result, Dempster-Shafer theory of evidence is applied to integrate the different forms of evidence.

### Data

Three kinds of data were used in this study: genetic sequences, reported H5N1 outbreak records, and socio-environmental factors, including altitude, population density, poultry density, and the shortest path distances to inland water, coastlines, migratory bird pathways, railways, and roads (see Figure S2). All these data were collated for lattices based on different spatial resolutions: 8.4 km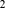, 34.22 km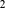, 0.94 km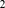, 0.32 km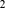, and 0.24 km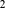 for East-Southeast Asia, China, Indonesia, Thailand, and Vietnam, respectively.

First, from GenBank [31], 888 genetic sequences of influenza A H5N1 hemagglutinin (HA) and neuraminidase (NA) genes were isolated from a variety of hosts between 1996 and 2009 across the areas of East-Southeast Asia covering Thailand, Vietnam, Laos, Cambodia, Indonesia, and China. With reported geographic localities, the sequences could be assigned to lattices by geocoding. Multiple alignment of all sequences from each HA and NA gene was carried out using MUSCLE [32], and the HA and NA sequences were combined using TaxonDNA [33]. A phylogenetic tree for the combined dataset was constructed using neighbor-joining (NJ) [34] in PAUP* 4.0 [35](see Figure S3). The best-fit model of genomic RNA substitution for the NJ analysis was assessed by Model test version 3.7 [36]. Base composition and pairwise comparisons were examined using MEGA version 4.02 [37]. The NJ algorithm was adopted in this study because this distance-based method of phylogenetic reconstruction gave the genetic distances among the sequences that needed to be combined with the results of the other analysis in the knowledge fusion step.

Second, outbreaks of the disease were assigned to lattice points. Data on avian influenza H5N1 outbreaks in East-Southeast Asia include 2204 avian and 327 human H5N1-confirmed cases between May 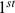 1997 to March 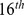 2009, compiled by the World Organisation of Animal Health (OIE, www.oie.int) and the World Health Organization (WHO, www.who.int), respectively. Each record contained the following attributes: country, province, location (latitude, longitude), start time, affected species, and the number of deaths. Using latitude and longitude, any outbreak could be assigned to the lattice. For each lattice point, the numbers of outbreaks of both human and avian influenza were recorded.

Third, the socio-environmental data were compiled for the raster grids based on the spatial resolutions of East-Southeast Asia, Indonesia and China, respectively. Poultry census data in 2005 were obtained from Food and Agriculture Organization's Animal Production and Health Division (FAO-AGA).

These poultry data, collected from sub-national livestock census data and corresponding to administrative boundaries, were converted into densities by excluding the areas unsuitable for livestock. The poultry density data were downloaded from GeoNetwork (www.fao.org/geonetwork/srv/en), and collated to raster grids with each pixel value representing actual density per km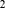. Population density was chosen as an indicator of the volume of viral traffic [13]. The 2010 estimate of population density, produced by the Center for International Earth Science Information Network (CIESIN), Columbia University and the United Nations Food and Agriculture Programme (FAO), were downloaded from the CIESIN (www.ciesin.org). Railways and roads were chosen because these two variables were used as surrogate indicator of long-range movements of human and poultry. The shape data for the two transport networks were provided by GIVA-GIS (www.diva-gis.org), an open source for mapping and geographic data. The area and line-based data, including inland water bodies, coastline, and migratory bird pathways, downloaded from the GIVA-GIS, were used to determine the association between birds and the outbreaks. The migratory bird pathways were specified by 70 km buffers on each side because of uncertainty about the behavior of migrating birds. All shapes were converted to binary lattice data indicating presence or absence. In addition average elevation data [90_m resolution Digital Terrain Model from the Shuttle Radar Topography Mission data, STRM V3 (http://srtm.csi.cgiar.org)] were used to capture topographic features that might be associated with the establishment of an H5N1 epidemic [13].

### Quantifying Phylogenetic Tree

In the phylogenetic trees of the H5N1 virus (Figures S3 and S4), the process of viral evolution is composed of a set of branches, originating from a common ancestor at the root of the tree. The length of a branch describes an evolutionary stage which starts from a previous hypothesized ancestor (or a node). The taxa having a common node can be regarded as a subgroup (or a clade). By this means, a phylogenetic tree is usually divided and the taxa are grouped into different subgroups, with the members of each subgroup are phylogenetically close. In Virology, subgroups are usually determined by eye balling [2], [8]. A subgroup is believed to have a strong capability of surviving if its members show a pattern of wide spatial dispersion and extensive persistence over time [3]. This also indicates the capability of a subgroup to spread the disease. However, for a virus, this capability can be measured by both the process of the viral evolution and the capability of the subgroups which the virus belongs to. The longer is the evolutionary process the greater is the ability of a virus to survive and the higher is the possibility of the spread of the virus. Also, a virus is believed to have a large capability if it is a member of a strong subgroup of the strains showing extensive persistence in space and time. For evaluation, it is necessary to sum up all evolutionary stages that a virus goes through. Each stage contains a branch and a node which is shared by a subgroup of the taxa. As a first approximation, 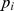, an estimate of the capability of a virus, can be measured by a linear sum,
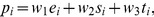
(1)where 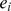, 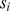, and 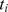 are viral evolution, spatial dispersion, and time span at a stage *i*. 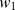, 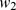, and 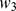 are weights for the three variables, assigned the values 0.4, 0.4, and 0.2, respectively. The lower weight for the time span is because a temporal scale of a year is coarse relative to the other two variables. The normalized values of 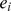, 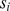, and 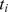 can be estimated from the hierarchical structure of the tree. First, the length of the branch at a stage *i* is used to measure viral evolution. Second, we measure how widely dispersed, geographically, members of the same subgroup are. Spatial dispersion is estimated by the total inertia (sum of the variances) of the 2
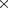
2 covariance matrix calculated using the locations (latitude, longitude) of the members at the same stage. Third, the length of time for a subgroup is measured by the time span from early to late occurrences of virus members. The time span 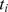 is a ratio, including the persistence of a subgroup at stage *i*. It is calculated as the time length *i* divided by the time length of the whole tree. Estimates of *e* and *s* are normalized to avoid the effect of large values. Mathematically, the capability of a virus can be evaluated by
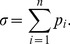
(2)where *n* is the number of evolutionary stages of the virus. Quantifying a phylogenetic tree enables us to estimate the capability of a virus.

To determine the range of a virus, we also need to examine the density of surrounding outbreaks at a series of scales from 10 to 250 km. Within this range, a scale *l* at which outbreak density is at a maximum was selected. The area affected by a virus is estimated using a spatial Gaussian kernel function:
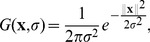
(3)If 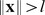 and 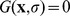, the range of the affected surface is *l*; otherwise, if 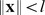 and 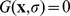, the range is 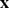. Figures 1(a) and (c) show the capability of H5N1 in Thailand and Vietnam based on quantifying the phylogenetic trees of the H5N1 virus (Figures S3(a) and (b)). The quantification of the trees of Indonesia, China, and East-Southeast Asia (Figures S3 and S4(c) and (d)) can be cross-referenced with to Figure S5(a), (d), and (g).

### Cluster Analysis

The local *K* function, first prosed by Getis et al. [38]–[40], is a statistic of spatial point patterns [30]. In this study, we apply a weighted local *K* function for the identification of clusters of outbreaks on lattices. The modified function can estimate the intensity of aggregation (for an example, see Figure S6) by taking into account the spatial effects arising from the locations of outbreaks. Specifically, this statistic weights the number of outbreaks by distance and can be expressed as 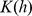 which equals the expected weighted number of outbreaks within distance *h* of a lattice point *i*. Mathematically, the estimate of the modified function is:
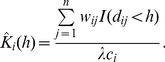
(4)where 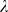 is the global density of outbreaks and 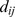 is the distance between lattice points *i* and *j*. *I* is an indicator function, commonly defined as 1 for 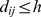 and 0 for 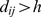. It indicates the outbreaks surrounding the lattice point *i* that are within a distance range *h*. Each such outbreak is weighted by the inverse Euclidean distance, 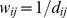. In this study, the modified local *K* function analysis set *h* = 113 km which is recommended by [41] as the appropriate scale for exploring local patterns of influenza.

The estimates obtained from this analysis can be used as predictors of the occurrence of H5N1 by normalizing. Figure 1(b) and (d), for instance, show the local patterns of the outbreak clusters in Thailand and Vietnam. The cluster analysis results of Indonesia, China, and East-Southeast Asia are shown in Figures S5(b), (e), and (h).

### Risk Factor Analysis

A logistic regression was fitted to model the association between the occurrence of H5N1 outbreaks and eight socio-environmental factors in East-Southeast Asia, Indonesia, and China. Data on the predictors were compiled for each lattice point, and the outbreaks converted to ‘presence of outbreaks’ (1) or ‘absence of outbreaks’ (0) for each lattice point.

A very large majority of lattice points have no outbreaks which makes fitting the logistic model to all the lattice points unsatisfactory (see also [13]). We build models by selecting all outbreak-present lattice points and randomly selecting double the number of lattices points with no outbreaks. The number of H5N1 outbreaks in East-Southeast Asia including Indonesia and China are much lower than in Thailand and Vietnam. One thousand bootstrap replicates were implemented to ensure a satisfactory sample size for carrying out model inference. In each repeat, the coefficient and 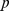 value of each predictor were estimated. Cohen's kappa index was used to evaluate the observed/model predicted misclassification matrix and Nagelkerke/Cragg & Uhler's psuedo-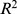 was used as the goodness-of-fit statistic for the logistic regression models. In addition, the receiver-operating characteristic ROC provides a two-dimensional depiction of predictive performance [42]. The area under the ROC curve (AUC) measures the probability of a correct classification [43]( Figure S7). The ROC, Cohen's kappa index, and Nagelkerke/Cragg & Uhler's psuedo-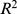 were calculated for the purpose of assessing the predictive power of the model (Table S1). All these estimates were averaged over the 1000 bootstrap replicates. The predictions for the H5N1 in Indonesia, China, and East-Southeast Asia are shown in Figures S5(c), (f), and (i).

### Knowledge Fusion and the Dempster-Shafer Theory of Evidence

Knowledge fusion provides a framework for integrating information from different research domains. Uncertainty, perhaps arising from incomplete data, limited domain knowledge, or the application of an insufficiently sophisticated methodology, could limit the value of the above analyses. Dempster-Shafer (D-S) theory of evidence seeks to overcome the limitations associated with conventional probability theory when the researcher seeks to quantify and reason with imprecise, uncertain and/or weak information [24], [25]. It has been widely used in applications involving the use of geographical information systems [44] and image processing technologies [45], [46]; it has also been used in climate change research [47], detecting credit card fraud [48], and in evaluating outcomes associated with medical intervention [49].

Recently, this method has been employed to identify areas at risk from rift valley fever in Africa [50]. To provide a formal description of D-S theory, 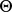 defines a finite set of mutually exclusive and exhaustive elementary hypothesis 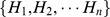 called the frame of discernment (*FOD*). The number of possible subsets of 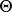 is 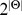, including the full and null hypotheses. In this study, each lattice point is a binary frame of a discernment containing two elementary hypotheses: 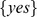 for the presence of H5N1 risk and 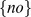 for absence, i.e., 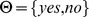. The subsets of 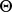 are 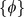, 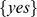, 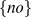, and 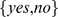. In particular, the subset 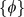 and 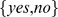 stand for empty and unknown (or uncertainty).

In D-S theory, each subset is assigned a belief value by the available evidence, called probability mass function, 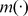. In particular, 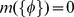, 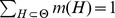, where 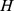 represents the subsets 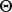 and 0
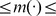
1. In this study, the results of the three analyses (the capability measure of the H5N1 virus, the clusters of outbreaks, and the results from the logistic regression analysis) provide evidence indicating the belief for the risk of occurrence of H5N1, denoted by 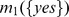, 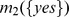, and 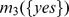. However, no evidence is directly provided by the first two studies regarding the absence of the disease. Therefore, the values of 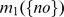 and 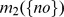 cannot be determined and so the mass functions of unknown, i.e. 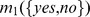 and 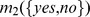, are assigned: 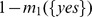 and 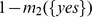. The logistic regression model results are different however because the predictive power of the model allows us to assign values to the mass functions. The average AUC is used to assess model predictions. The multiplication of the predicted value and the average AUC is the evidence for 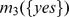. The values of 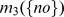 and 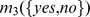 are thus 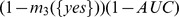 and 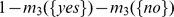, respectively.

The Dempster's rule of combination offers an approach to combining evidence from different sources. The joint probability mass function, for instance 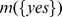, can be obtained from the combination of the two mass functions, 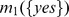 and 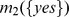:
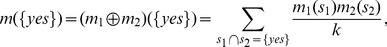
(5)where
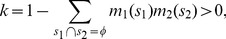
(6)where *k* is a normalization factor, and 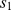 and 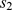 are subsets of 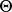. Dempster's rule is commutative and associative, and thus the joint mass function is independent of the order in which evidence are combined [24].

In this study, the three sources of evidence are combined via an iterative procedure in order to identify for each lattice point the ‘degree of belief’ we have in the likely occurrence of H5N1. To simplify the text we have referred to this as the “risk”. Thailand, for example, is represented by a matrix 3088
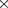
1738 lattice points. Figure S1(c) shows the integrated result of the H5N1 epidemic in Thailand. Dempster's combination procedure is demonstrated for Thailand in Table S2.

### Spatial Correspondence Analysis

Spatial correspondence analysis using Pearson's correlation coefficient, 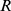, [51], [52] measures the association between observed avian-influenza outbreaks and model predictions. A sample size adjusted t-test was employed to test statistical significance of 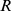. The reduction in the degrees of freedom is a function of the level of spatial autocorrelation in the two maps that are being correlated. This is because spatial autocorrelation introduces redundancy into a set of data and the adjustment procedure identifies the “equivalent” number of independent observations.

For the implementation, lattices were aggregated into blocks varying in size from 60
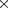
60 to 30
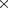
30 because the number of cases is small relative to the original spatial resolution of the data which is extremely fine-grained. The observed outbreaks were converted to a rate by calculating for each areal unit the number of outbreak cases divided by the population at risk which is the human plus poultry population, (number of outbreak)/(population
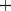
poultry). Model predictions were obtained by averaging results across the lattice points falling within any block. Results from the analysis of spatial correspondence using the sample size adjusted t-test for significance testing are shown in Figure S1.

## Supporting Information

Figure S1 **Test for spatial correspondence of H5N1 outbreaks and the empirical patterns.**
(TIF)

Figure S2 **Spatial distribution of H5N1: spatial distribution of reported H5N1 outbreaks, inland water bodies, migratory bird pathways, pathways, and roads in East-Southeast Asia.**
(TIFF)

Figure S3 **NJ tree of the 888 H5N1 concatenation of hemagglutinin (HA) and neuraminidase (NA) DNA sequences from East-Southeast Asia covering Thailand, Vietnam, Cambodia, Laos, Indonesia, and China.** The best model is TVM+I+G (transversional model incorporating invariable sites and rate variation among sites). The goose H5N1 DNA sequence from Guangdong in 1996 (A/Goose/Guangdong/1/96) is used to root the tree. The length of a unit is .02. The taxa are colored by locality with green for Thailand, blue for Vietnam, orange for Indonesia, and black for all others including China, Cambodia, Laos, and purple, especially, for Qinghai province, western China.
(TIF)

Figure S4 **NJ trees for the H5N1 virus from (a) Thailand, (b) Vietnam, (c) Indonesia, and (d) China.** The best model is GTR+I+G (General Time Reversible incorporating invariable sites and rate variation among sites.
(TIF)

Figure S5 **Results from phylogenetic tree analysis, modified local** ***K*** **function analysis, and logistic regression analysis for Indonesia, China, East-Southeast Asia.** The values shown in the color bar are the probability of outbreaks of H5N1. (a), (d), (g) are the outcomes from the phylogenetic tree analysis (Figure 3(b) and Figures S3(c) and (d)), showing the spatial profile of the capability of the H5N1 virus; (b), (e), and (h) are the results from the modified local *K* function analysis, depicting the spatial distribution of outbreak clusters; (c), (f), and (i) show the predictive results from the logistic regression models.
(TIF)

Figure S6 **Illustration of the original and modified local** ***K*** **function.** *Note*: the original local *K* function based on the number of outbreaks cannot distinguish between the cluster with the red and blue circles. The modified local *K* function, by taking into account the distance between outbreaks, is able to distinguish the two patterns.
(TIF)

Figure S7 **ROC curves for the logistic regression model.** The area under the ROC curve (AUC) indicates the probability of a correct classification. (a) and (b) show the average AUC of the models for outbreaks of East-Southeast Asia and Indonesia from 1996 to 2009. (c), (d), (e) are the AUC of the models for China for the periods between 1996 and 2009, 1996 and 2004, and 2005 and 2009, respectively. The blue areas are the envelopes of the 1000 bootstrap replicates.
(TIF)

Table S1 Logistic regression model assemssment for the H5N1 occurrences in East-Southeast Asia, Inodnesia, and China, 1996–2009 and the two epidemic waves between 1996–2004 and 2005–2009.
(PDF)

Table S2 Dempster's combination for the three sources of evidence on H5N1 in Thailand.
(PDF)

## References

[1] Xu X, Subbarao K, Cox NJ, Guo Y (1999). Genetic characterization of the pathogenic influenza A/Goose/Guangdong/1/96 (H5N1) virus: Similarity of its Hemagglutinin gene to those of H5N1 viruses from the 1997 outbreaks in Hong Kong.. Virology.

[2] Li KS, Guan Y, Wang J, Smith GJD, Xu KM (2004). Genesis of a highly pathogenic and potentially pandemic H5N1 influenza virus in eastern Asia.. Nature.

[3] Chen H, Smith GJD, Li KS, Wang J, Fan XH (2006). Establishment of multiple sublineages of H5N1 influenza virus in Asia: Implications for pandemic control.. Proc Natl Acad Sci U S A.

[4] Enserink M (2006). H5N1 moves into Africa, European Union, deepening global crisis.. Science.

[5] WHO (2010). Cumulative number of confirmed human cases of avian influenza A/(H5N1).. http://www.who.int/csr/disease/avian.

[6] Preiser KH (2006). Influenza Report 2006, Waye MY, editor.

[7] Dawood F, Jain S, Finelli L, Shaw M, Lindstrom S (2009). Emergence of a novel swine-origin influenza A (H1N1) virus in humans.. N Engl J Med.

[8] Smith GJD, Naipospos TSP, Nguyen TD, de Jong MD, Vijaykrishna D (2006). Evolution and adaptation of H5N1 influenza virus in avian and human hosts in Indonesia and Vietnam.. Virology.

[9] Duan L, Campitelli L, Fan XH, Leung YHC, Vijaykrishna D (2007). Characterization of low-pathogenic H5 subtype influenza viruses from eurasia: Implications for the origin of highly pathogenic H5N1 viruses.. J Virol.

[10] Avise JC, Arnold J, Ball RM, Bermingham E, Lamb T (1987). Intraspecific phylogeography: The mitochondrial DNA bridge between population genetics and systematics.. Annu Rev Ecol Syst.

[11] Wallace RG, Hodac H, Lathrop RH, Fitch WM (2007). A statistical phylogeography of influenza A H5N1.. Proc Natl Acad Sci U S A.

[12] Si Y, Skidmore AK, Wang T, de Boer WF, Debba P (2009). Spatio-temporal dynamics of global H5N1 outbreaks match bird migration patterns.. Geospatial Health.

[13] Gilbert M, Xiao X, Pfeiffer DU, Epprecht M, Boles S (2008). Mapping H5N1 highly pathogenic avian influenza risk in southeast Asia.. Proc Natl Acad Sci U S A.

[14] Openshaw S (1984). The Modifiable Areal Unit Problem.

[15] Fang LQ, de Vlas SJ, Liang S, Looman CWN, Gong P (2008). Environmental factors contributing to the spread of H5N1 avian influenza in mainland China.. PLoS ONE.

[16] FAO (2010). Technical task force on avian influenza. Update on the avian influenza situation.. http://www.fao.org/avianu/en/aidenews.html.

[17] Smith GJD, Fan XH, Wang J, Li KS, Qin K (2006). Emergence and predominance of an H5N1 influenza variant in China.. Proc Natl Acad Sci U S A.

[18] Smallman-Raynor M, Cliff AD (2008). The geographical spread of avian influenza A (H5N1): Pan- zootic transmission (December 2003–May 2006), pandemic potential, and implications.. Ann Assoc Am Geogr.

[19] Cavalli-Sforza LL, Edwards AWF (1967). Phylogenetic analysis: Models and estimation procedures.. Am J Hum Genet.

[20] Penny D, Hendy MD, Steel MA (1992). Progress with methods for constructing evolutionary trees.. Trends Ecol Evol.

[21] Kilpatrick AM, Chmura AA, Gibbons DW, Fleischer RC, Marra PP (2006). Predicting the global spread of H5N1 avian influenza.. Proc Natl Acad Sci U S A.

[22] Liang L, Xu B, Chen Y, Liu Y, Cao W (2010). Combining spatial-temporal and phylogenetic analysis approaches for improved understanding on global H5N1 transmission.. PLoS ONE.

[23] Carrel M, Emch M, Jobe R, Moody A, Wan X (2010). Spatiotemporal structure of molecular evolution of H5N1 highly pathogenic avian influenza in Vietnam.. PLoS ONE.

[24] Dempster AP (1967). Upper and lower probabilities induced by a multivalued mapping.. Ann Math Stat.

[25] Shafer G (1976). A Mathematical Theory of Evidence.

[26] Wan XF, Nguyen T, Davis CT, Smith CB, Zhao ZM (2008). Evolution of highly pathogenic H5N1 avian influenza viruses in Vietnam between 2001 and 2007.. PLoS ONE.

[27] Chen H, Smith GJD, Zhang SY, Qin K, Wang J (2005). Avian u: H5N1 virus outbreak in migratory waterfowl.. Nature.

[28] Liu J, Xiao H, Lei F, Zhu Q, Qin K (2005). Highly pathogenic H5N1 influenza virus infection in migratory birds.. Science.

[29] Olsen B, Munster VJ, Wallensten A, Waldenstrm J, Osterhaus ADME (2006). Global patterns of influenza a virus in wild birds.. Science.

[30] Botts BN, Getis A (1988). Point Pattern Analysis.. Scientific geography series, Vol. 8.

[31] Benson DA, Karsch-Mizrachi I, Lipman DJ, Ostell J, Wheeler DL (2006). GenBank.. Nucleic Acids Res.

[32] Edgar R (2004). MUSCLE: Multiple sequence alignment with high accuracy and high throughput.. Nucleic Acids Res.

[33] Meier R, Kwong S, Vaidva G, Ng P (2006). DNA barcoding and taxonomy in Diptera: A tale of high intraspecific and low identification succeess.. Syst Biol.

[34] Saitou N, Nei M (1987). The neighbor-joining method: A new method for reconstructing phyloge- netic trees.. Mol Bio Evol.

[35] Swofford D (2002). PAUP*: Phylogenetic Analysis Using Parsimony (* and other methods).

[36] Posada D, Crandall K (1998). MODELTEST: Testing the model of DNA substitution.. Bioinformatics.

[37] Tamura K, Dudley J, Nei M, Kumar S (2007). MEGA4: Molecular evolutionary genetics analysis (MEGA) software version 4.0.. Mol Bio Evol.

[38] Getis A (1984). Interaction modeling using second-order analysis.. Environ Plann A.

[39] Getis A, Franklin J (1987). Second-order neighborhood analysis of mapped point patterns.. Ecology.

[40] Getis A, Ord JK (1996). Local Spatial Statistics: An overview.

[41] Viboud C, Bjornstad ON, Smith DL, Simonsen L, Miller MA (2006). Synchrony, waves, and spatial hierarchies in the spread of influenza.. Science.

[42] Fawcett T (2006). An introduction to ROC analysis.. Pattern Recogn Lett.

[43] Hanely J, McNeil B (1982). The meaning and use of the area under a receiver operating characteristic (ROC) curve.. Radiology.

[44] Malpica J, Alonso M, Sanz M (2007). Dempster-Shafer theory in geographic information systems: A survey.. Expert Syst Appl.

[45] Kaftandjian V, Dupuis O, Babot D, Zhu YM (2003). Uncertainty modelling using Dempster-Shafer theory for improving detection of weld defects.. Pattern Recogn Lett.

[46] Adamek T, O'Connor N (2007). Using Dempster-Shafer theory to fuse multiple information sources in region-based segmentation.. http://dx.doi.org/10.1109/ICIP.2007.4379144.

[47] Luo WB, Caselton B (1997). Using Dempster-Shafer theory to represent climate change uncertainties.. J Environ Manage.

[48] Panigrahi S, Kundu A, Sural S, Majumdar A (2009). Credit card fraud detection: A fusion approach using Dempster-Shafer theory and Bayesian learning.. Inform Fusion.

[49] Jones L, Beynon MJ, Holt CA, Roy S (2006). An application of the Dempster-Shafer theory of evidence to the classification of knee function and detection of improvement due to total knee replacement surgery.. J Biomec.

[50] Clements A, Pfeiffer D, Martin V (2006). Application of knowledge-driven spatial modelling approaches and uncertainty management to a study of Rift Valley fever in Africa.. Int J Health Geogr.

[51] Haining R (1991). Bivariate correlation with spatial data.. Geogr Anal.

[52] Haining R (2003). Spatial Data Analysis: Theory and Practice.

