# Supplementary figures and images for: Using Knowledge Fusion to Analyze Avian Influenza H5N1 in East and Southeast Asia
Source: PLoS One. 2012 May 17;7(5):e29617. doi: 10.1371/journal.pone.0029617 (PMC3355188; doi:10.1371/journal.pone.0029617)

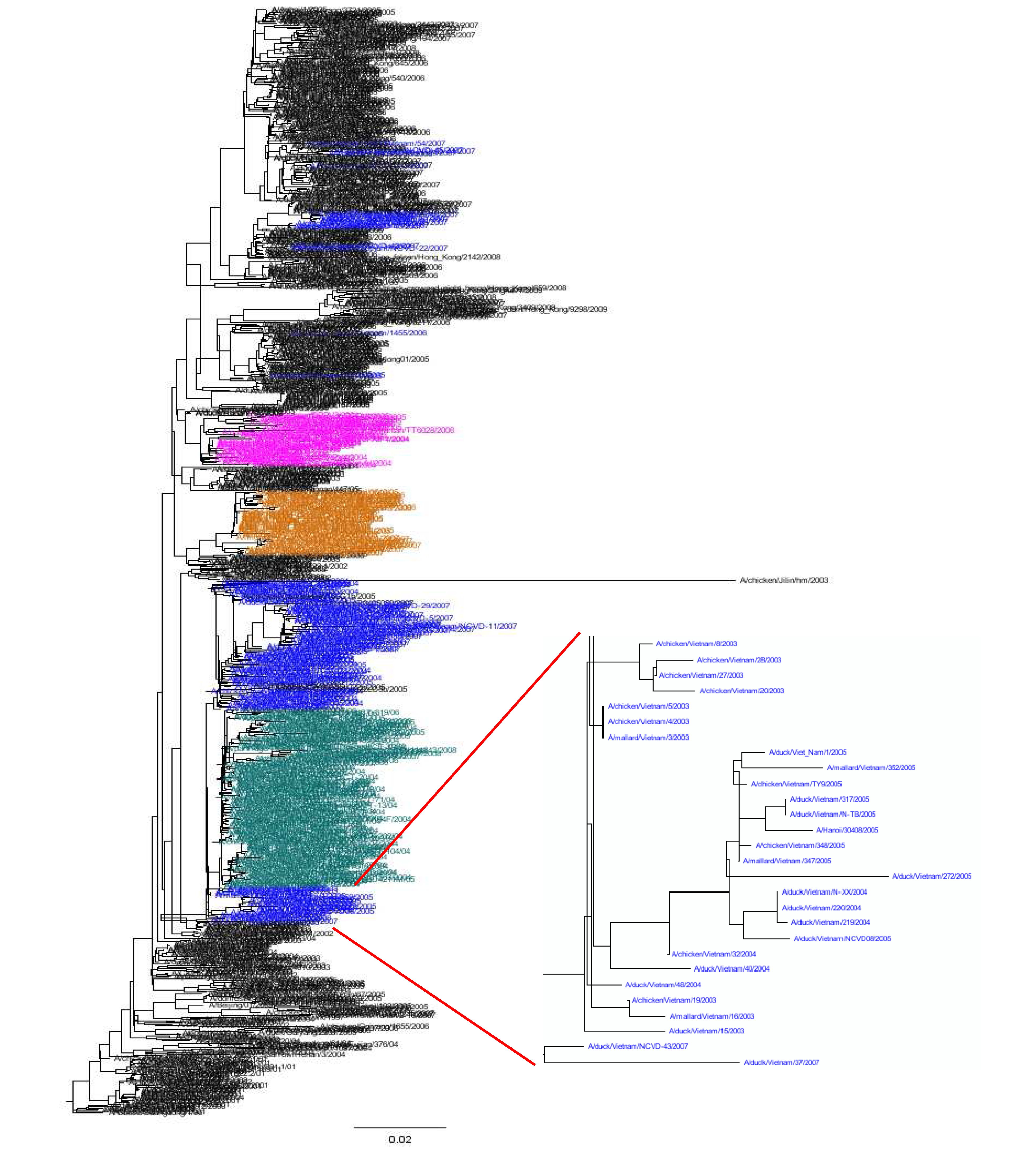

Supplement: Figure S3 — NJ tree of the 888 H5N1 concatenation of hemagglutinin (HA) and neuraminidase (NA) DNA sequences from East-Southeast Asia covering Thailand, Vietnam, Cambodia, Laos, Indonesia, and China. The best model is TVM+I+G (transversional model incorporating invariable sites and rate variation among sites). The goose H5N1 DNA sequence from Guangdong in 1996 (A/Goose/Guangdong/1/96) is used to root the tree. The length of a unit is .02. The taxa are colored by locality with green for Thailand, blue for Vietnam, orange for Indonesia, and black for all others including China, Cambodia, Laos, and purple, especially, for Qinghai province, western China. (TIF) [file pone.0029617.s003.tif]

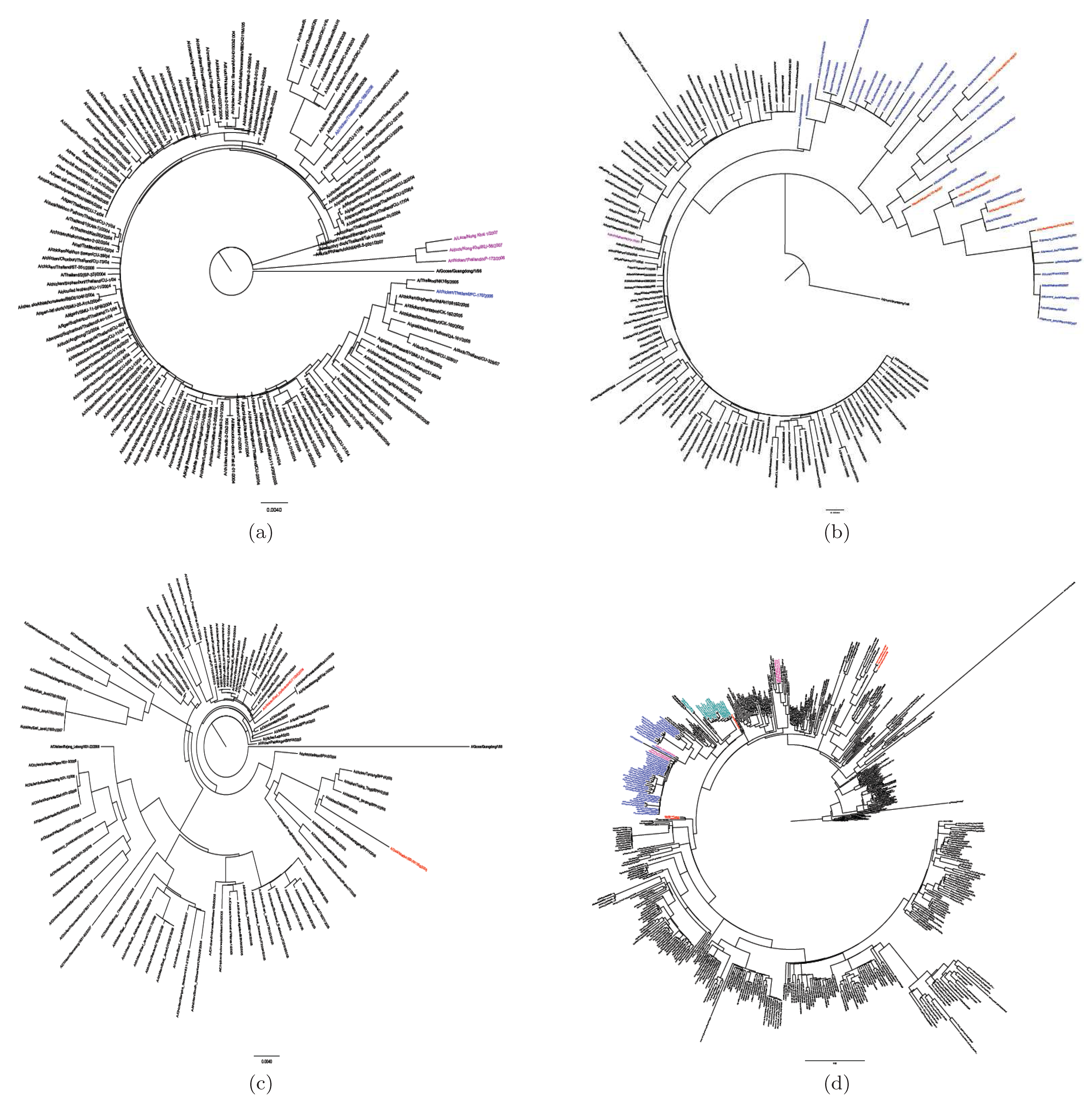

Supplement: Figure S4 — NJ trees for the H5N1 virus from (a) Thailand, (b) Vietnam, (c) Indonesia, and (d) China. The best model is GTR+I+G (General Time Reversible incorporating invariable sites and rate variation among sites. (TIF) [file pone.0029617.s004.tif]

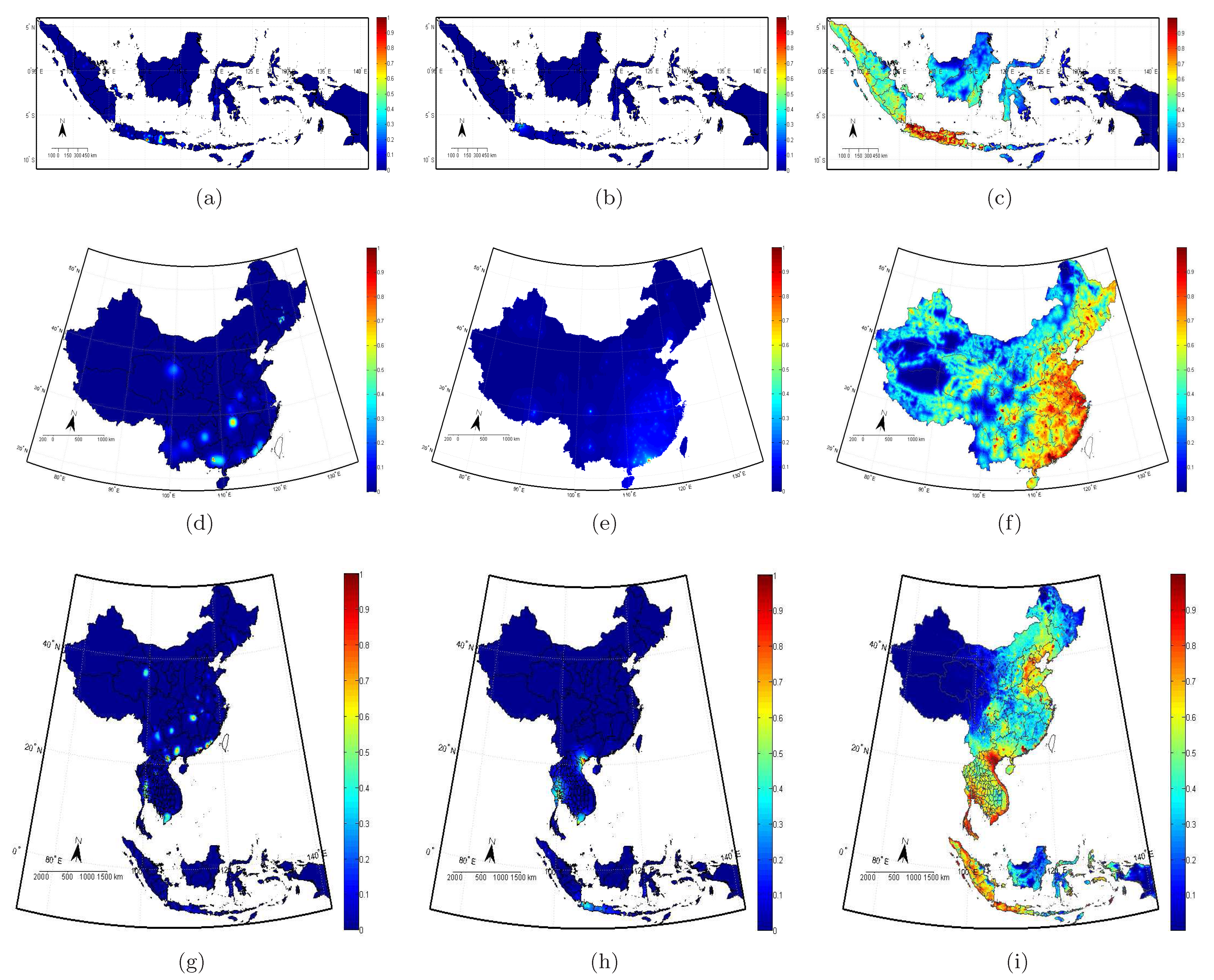

Supplement: Figure S5 — Results from phylogenetic tree analysis, modified local K function analysis, and logistic regression analysis for Indonesia, China, East-Southeast Asia. The values shown in the color bar are the probability of outbreaks of H5N1. (a), (d), (g) are the outcomes from the phylogenetic tree analysis (Figure 3(b) and Figures S3(c) and (d)), showing the spatial profile of the capability of the H5N1 virus; (b), (e), and (h) are the results from the modified local K function analysis, depicting the spatial distribution of outbreak clusters; (c), (f), and (i) show the predictive results from the logistic regression models. (TIF) [file pone.0029617.s005.tif]

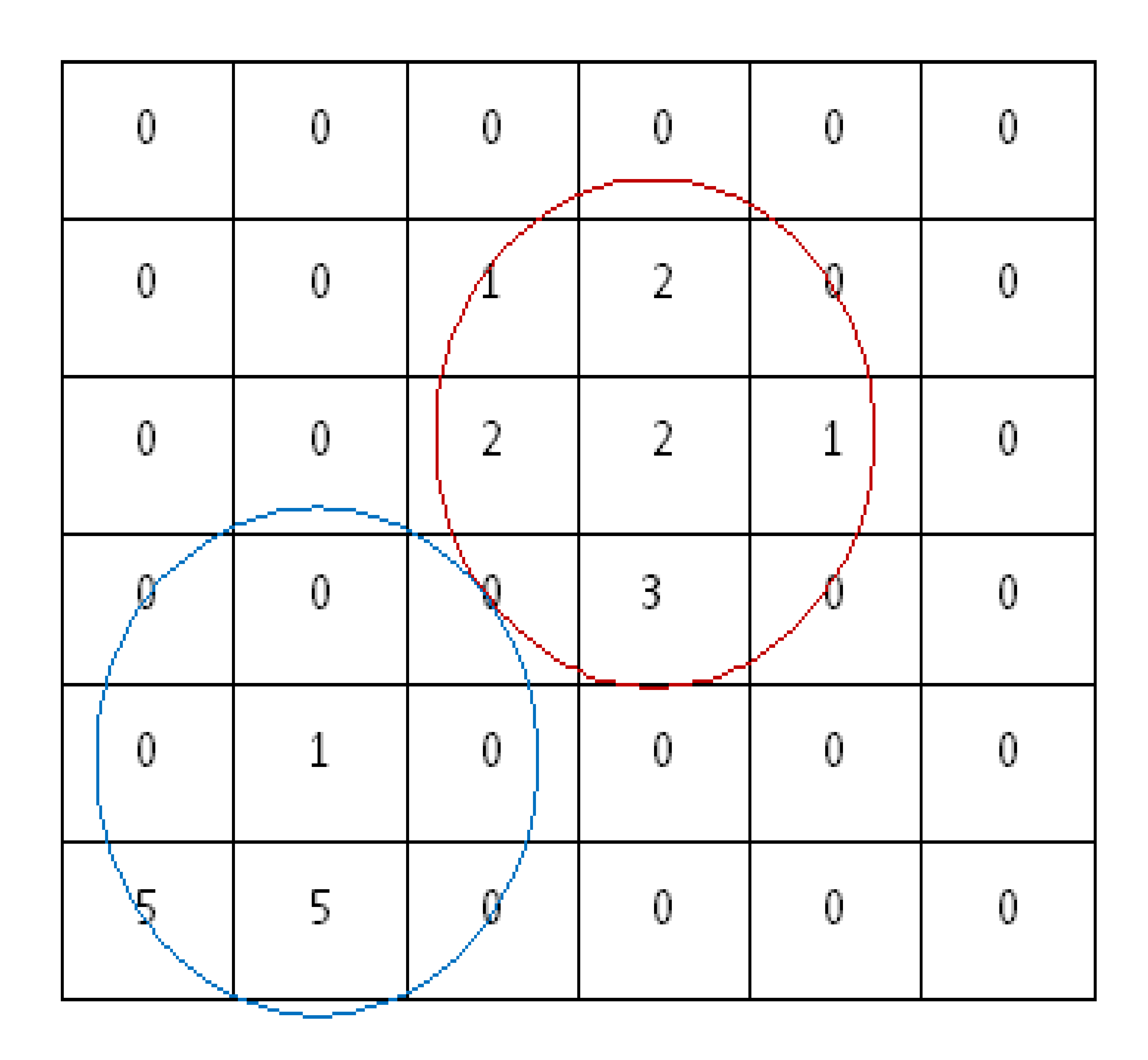

Supplement: Figure S6 — Illustration of the original and modified local K function. Note: the original local K function based on the number of outbreaks cannot distinguish between the cluster with the red and blue circles. The modified local K function, by taking into account the distance between outbreaks, is able to distinguish the two patterns. (TIF) [file pone.0029617.s006.tif]
