# Supplementary material for: Using Knowledge Fusion to Analyze Avian Influenza H5N1 in East and Southeast Asia
Source: PLoS One. 2012 May 17;7(5):e29617. doi: 10.1371/journal.pone.0029617 (PMC3355188; doi:10.1371/journal.pone.0029617)
Supplement: Table S1 — Logistic regression model assemssment for the H5N1 occurrences in East-Southeast Asia, Inodnesia, and China, 1996–2009 and the two epidemic waves between 1996–2004 and 2005–2009. (PDF) [file pone.0029617.s008.pdf]

Table S1 Logistic regression model assessment for the H5N1 occurrences in East-Southeast Asia, Indonesia, and China, 1996-2009 and the two epidemic waves between 1996-2004 and 2005-2009.

	Model Assessment		
Region or Country	AUC±SD	Kappa±SD	Pseudo-R2±SD
East-Southeast Asia	.9034*±.0019	8467±.0130	.6061±.0060
Indonesia	.8947±.0044	.6491±.0042	.4751±.0192
China (1996-2009),	.7806±.0097	.6063±.0408	.2354±.0241
China (1996-2004),	.8865±.0180	.6497±.0181	.4901±.0681
China (2005-2009),	.7221±.0132	.5796±.1021	.1679±.0269

* Average value of 1000 logistic models
