# Supplementary material for: Using Knowledge Fusion to Analyze Avian Influenza H5N1 in East and Southeast Asia
Source: PLoS One. 2012 May 17;7(5):e29617. doi: 10.1371/journal.pone.0029617 (PMC3355188; doi:10.1371/journal.pone.0029617)
Supplement: Table S2 — Dempster's combination for the three sources of evidence on H5N1 in Thailand. (PDF) [file pone.0029617.s009.pdf]

Table S2 Dempster's combination for the three sources of evidence on H5N1 in Thailand.

	Lattice Point Locations (Row: 1330, Column: 621-630)									
Evidence	621	622	623	624	625	626	627	628	629	630
$m_1(\{yes\})$	.5920	.6144	.6357	.6558	.6747	.6922	.7081	.7222	.7345	.7447
$m_1(\{no\})$	.0	.0	.0	.0	.0	.0	.0	.0	.0	.0
$m_1(\{yes, no\})$	.4080	.3856	.3643	.3442	.3253	.3078	.2919	.2778	.2655	.2553
$m_2(\{yes\})$	.6623	.6650	.6678	.6709	.6743	.6765	.6810	.6864	.6927	.7002
$m_2(\{no\})$	.0	.0	.0	.0	.0	.0	.0	.0	.0	.0
$m_2(\{yes, no\})$	.3377	.3350	.3322	.3291	.3257	.3235	.3190	.3136	.3073	.2998
$m_3(\{yes\})$	.6300	.6304	.5211	.3687	.3687	.3682	.5207	.5953	.6559	.6562
$m_3(\{no\})$	.1110	.1109	.1437	.1894	.1894	.1895	.1438	.1214	.1032	.1031
$m_3(\{yes, no\})$	.2590	.2587	.3352	.4419	.4419	.4423	.3355	.2833	.2409	.2407
Combination										
$m(\{yes\})$	.9436	.9472	.9337	.9141	.9195	.9241	.9487	.9604	.9690	.9709
$m(\{no\})$	.0169	.0159	.0199	.0258	.0242	.0228	.0154	.0119	.0093	.0087
$m(\{yes, no\})$	.0395	.0370	.0464	.0601	.0564	.0531	.0359	.0278	.0217	.0204
